# Cardiac Extracorporeal Membrane Oxygenation in Community Cardiac Surgery Program: Are the Results Comparable?

**DOI:** 10.7759/cureus.58947

**Published:** 2024-04-24

**Authors:** Syed Saif Abbas Rizvi, Matthew Nagle, Brian Roberts, Lydia McDermott, Kathleen Miller, Claudine Pasquarello, Anissa Braddock, Chun Choi, Qiong Yang, Hitoshi Hirose

**Affiliations:** 1 Surgery, Virtua Our Lady of Lourdes Hospital, Camden, USA; 2 Cardiovascular and Thoracic Surgery, Cleveland Clinic, Cleveland, USA

**Keywords:** patient outcomes, veno-arterial extracorporeal membrane oxygenation (va ecmo), extracorporeal cardiopulmonary resuscitation, community hospital, extracorporeal membrane oxygenation

## Abstract

Background: Extracorporeal membrane oxygenation (ECMO) outcomes in small centers are commonly considered less favorable than in large-volume centers. New ECMO protocols and procedures were established in our regional community hospital system as part of a cardiogenic shock initiative. This retrospective study aims to evaluate the outcomes of veno-arterial extracorporeal membrane oxygenation (VA ECMO) and extracorporeal cardiopulmonary resuscitation (ECPR) in a community hospital system with cardiac surgery capability and assess whether protocol optimization and cannulation standards result in comparable outcomes to larger centers whether the outcomes of this new ECMO program at the community hospital setting were comparable to the United States averages.

Methods: Our regional system comprises five hospitals with 1500 beds covering southwestern New Jersey, with only one of these hospitals having cardiac surgery and ECMO capability. In May 2021, the new ECMO program was initiated. Patients were screened by a multidisciplinary call, cannulated by our ECMO team, and subsequently treated by the designated team. We reviewed our cardiac ECMO outcomes over two years, from May 2021 to April 2023, in patients who required ECMO due to cardiogenic shock or as a part of extracorporeal cardiopulmonary resuscitation (ECPR).

Results: A total of 60 patients underwent cardiac ECMO, and all were VA ECMO, including 18 (30%) patients who required ECPR for cardiac arrest. The overall survival rate for our cardiac ECMO program turned out to be 48% (29/60), with 50% (22/42) in VA ECMO excluding ECPR and 39% (7/18) in the ECPR group. The hospital survival rate for the VA ECMO and ECPR groups was 36% (15/42) and 28% (5/18), respectively. The ELSO-reported national average for hospital survival is 48% for VA ECMO and 30% for ECPR. Considering these benchmarks, the hospital survival rate of our program did not significantly lag behind the national average.

Conclusions: With protocol, cannulation standards, and ECMO management optimized, the VA ECMO results of a community hospital system with cardiac surgery capability were not inferior to those of larger centers.

## Introduction

Extracorporeal membrane oxygenation (ECMO) serves as a pivotal form of temporary mechanical circulatory support (MSC) that can transform the prognosis for patients contending with refractory cardiac or respiratory failure [1]. Veno-arterial extracorporeal membrane oxygenation (VA ECMO) is a form of temporary life support, and when used in patients undergoing cardiopulmonary resuscitation during cardiac arrest, it is termed extracorporeal cardiopulmonary resuscitation (ECPR) [1]. By draining blood from the venous system, facilitating gas exchange, and then pumping it back into the major arterial vessels, VA ECMO delivers robust circulatory support to maintain end-organ perfusion. Compared to other mechanical support, such as percutaneous ventricular support or an intra-aortic balloon pump, VA ECMO is the only device to provide biventricular cardiac and respiratory support. Other benefits include the simplicity of the procedure and the fact that it does not necessarily require imaging modality while placing VA ECMO, which further expands the utility of VA ECMO during cardiopulmonary resuscitation.

To keep pace with the rising need for ECMO, high-volume ECMO centers have embraced a multidisciplinary approach and proven techniques aimed at preventing complications, enhancing survival rates, and improving overall patient outcomes [2-4]. However, despite numerous advancements in the field, the mortality and morbidity rates for ECMO patients persist at elevated levels [5,6]. Studies comparing mortality rates between high-volume and low-volume ECMO centers have produced variable results [7-9]. Yet, the overall data on the outcome and survival of patients in low-volume community hospitals remains scarce. Thus far, limited research has been conducted at community hospital centers regarding outcomes and survival rates for patients undergoing VA ECMO for cardiogenic shock or ECPR. However, veno-venous ECMO for respiratory failure has recently been used successfully at community hospitals during the coronavirus pandemic [10,11]. Considering the increasing prevalence of ECMO use in community hospitals, fostering effective communication and judicious resource utilization by implementing a multidisciplinary approach becomes paramount. These measures are crucial in influencing the survival outcomes of ECMO patients and achieving comparability to the national ECMO outcomes.

This article was previously presented as a meeting abstract at the 2023 American Society for Artificial Internal Organs (ASAIO) Annual Meeting on September 29, 2023

## Materials and methods

Our regional health system is comprised of five hospitals with a total of 1500 beds that cover southwestern New Jersey, though only one of these hospitals has the capability for cardiac surgery and ECMO cannulation. This central hospital is a community-based institution that is comprised of 350 beds, including a 21-bed cardiovascular intensive care unit (CVICU) and is registered in the Extracorporeal Life Support Organization (ELSO) center list (ID 1032).

**Figure 1:**
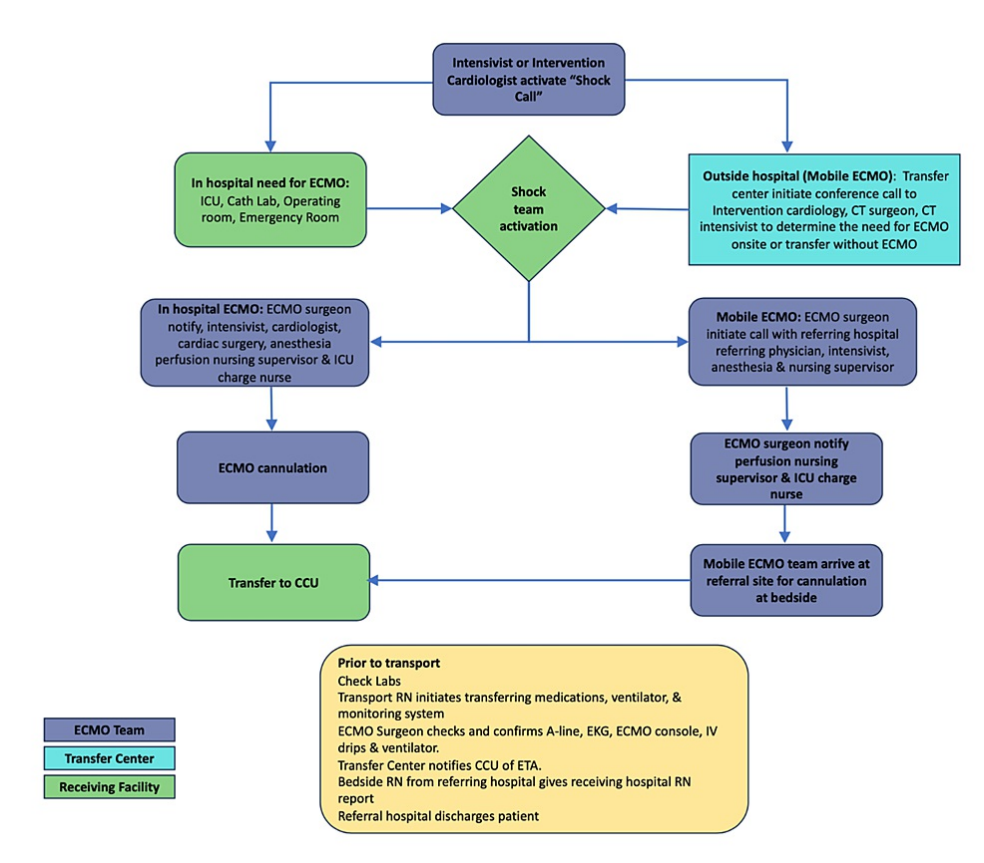
Process map of our extracorporeal membrane oxygenation process map The extracorporeal membrane oxygenation (ECMO) process map shows a flow diagram of our center's ECMO team activation and patient stabilization process. A cardiogenic shock (cardiac arrest or other cause of cardiogenic shock) call is activated by the physician at the bedside to our transfer center. The transfer center initiates a conference call with intervention cardiology, cardiothoracic surgeon, and cardiothoracic intensivist to determine the need for ECMO. This helped us to determine the urgency of initiating the ECMO onsite or after transferring the patient to our institution without ECMO. In the case of mobile ECMO, the ECMO cannulation team is transported by paramedics from the hub hospital to the local hospital, where they perform cannulation on the patient. The patient is transferred to the hub hospital for further care after this procedure. CCU: Critical care unit; ETA: Estimated time of arrival

In May 2021, we initiated our new ECMO program, created with the increasing need for ECMOs at our community hospital. We built a cannulation guideline [12] to meet the needs and demands of our institution and patient population. At our institution, the decisions to initiate cardiac ECMO are made through a multidisciplinary team conference call, which includes the heart failure cardiologist, interventional cardiologist, cardiothoracic surgeon, and cardiothoracic surgical intensivist (Figure 1). This helped us to determine the urgency of initiating the ECMO onsite or after transferring the patient to our institution without ECMO.

At our institution, patients were considered for cardiac ECMO initiation if they present with refractory cardiogenic shock unresponsive to maximal vasopressor and inotrope support or if they require cardiopulmonary resuscitation during cardiac arrest and do not respond to conventional CPR. Our protocol for excluding cardiac ECMO aligns with the guidelines set forth by the Extracorporeal Life Support Organization (ELSO) (Table 1). Absolute contraindications immediately disqualify a patient from ECMO, while relative contraindications prompt a detailed assessment and consensual decision by the multidisciplinary team on whether to proceed with ECMO. During the conference call, the decision to initiate pre-ECMO support was made by the referring cardiologist or cardiac surgeon in charge of the patient's care.

**Table 1:** Standard and relative contraindications to cardiac ECMO placement CPR: cardiopulmonary resuscitation; ECMO: extracorporeal membrane oxygenation; ROSC: return of spontaneous circulation

Standard contraindications
Age > 70
Body mass index > 45 with a high risk of vascular access
Mechanical ventilation > 7 days
Multiorgan failure
End-stage liver disease
Aortic dissection or severe aortic regurgitation
Irreversible neuro damage (previous stroke and neuro deficit)
Contraindications of anticoagulation
Cardiac arrest without ROSC for mobile ECMO
CPR more than 30 minutes at the time of consult for in-patient
Ongoing CPR without ROSC from outside of the hospital
Not accept transfusion
Active do-not-resuscitate order
Nothing to bridge (no ventricular assist device or transplant candidate)
No vascular access
Presence of inferior vena cava filter
Relative contraindications
Body mass index > 40
Septic shock
Severe commodities: severe chronic lung disease (home oxygen), Liver cirrhosis
Inability of access neuro status
No family or appropriate power of attorney
Presence of a mechanical valve

All cardiac ECMO patients underwent veno-arterial ECMO (VA ECMO) via cannulation to the femoral artery and vein, except for post-cardiotomy patients cannulated in the operating room. Peripheral ECMO cannulations were accompanied by a distal perfusion catheter to avoid distal limb ischemia. Central cannulation, which consisted of using the right atrium for drainage and ascending aorta for return, was used for post-cardiotomy patients who were unable to wean from cardiopulmonary bypass. ECMO cannulations were performed by the designated cardiothoracic surgeons or cardiothoracic surgical intensivists.

The ECMO pump used was either a Rotaflow pump (Getinge; Gothenburg, Sweden) or CentriMag pump (Abbott; Illinois, United States), both with a Quadrox-i oxygenator (Getinge; Gothenburg, Sweden). Urgent left ventricular vent using an Impella CP device (Abbott; Illinois, United States) was considered if the patient had a cardiac arrest under ECMO, minimum aortic pulse pressure on ECMO less than 10 mm Hg, or no aortic valve opening during cardiac systole. During ECMO, we maintained hemoglobin 8 g/dl. All VA-ECMO patients were managed in the CVICU under the supervision of cardiothoracic surgical intensivists.

We reviewed the electronic medical records of all patients at our institution placed on cardiac ECMO over two years, from May 2021 to April 2023. These patients were then divided into two groups: the non-ECPR VA ECMO group and the ECPR VA ECMO group. Data was collected retrospectively by reviewing the medical charts, which our hospital approved by the Institutional Review Board (certification # GLL11). The authors validated the data. Our study was exempt from patient consent due to the study being of retrospective analysis, and the patients were deidentified.

Baseline characteristics, demographics, ECMO survival, hospital survival, and survival to discharge were analyzed and reported using descriptive statistics in Excel and SPSS version 29. Data are presented as mean ± standard deviation (SD), mean ± standard error of the mean (SEM) (range), and median, interquartile range (IQR) for continuous variables, whereas percentages are used for categorical variables. A p-value of less than 0.05 was considered significant.

## Results

A total of 60 patients (45 males, 15 females, mean age of 59.4 +/- 12.9, range 24-77 years old) were placed on cardiac ECMO over two years. All of these were VA ECMO. No other form of ECMO, such as veno-venous ECMO, or veno-veno-arterial ECMO was used. The demographics are shown in (Table 2). The most common cause of patients requiring ECMO was acute myocardial infarction followed by post-cardiotomy (Table 3).

**Table 2:** Patient demographics of cardiac extracorporeal membrane oxygenation (values are expressed n (%), mean ± standard deviation, minimum – maximum) AKI: acute kidney injury; ECMO: Extracorporeal membrane oxygenation; ECPR: ECMO assisted Cardiopulmonary resuscitation; RIFEL: Risk, Injury, Failure, Loss, and End-stage kidney disease

Demographics	
Total number of Cardiac ECMO	60
Sex: Male	45 (75%)
Age, years	59.4 ± 12.9 (24-77)
Body surface area, m^2^	2.03 ± 0.24 (1.62 – 2.61)
Body mass index, kg/m^2 ^	30.7 ± 7.0 (18.7-56.0)
Mobile ECMO	9 (15%)
ECPR	18 (30%)
Pre ECMO Comorbidity	-
Smoking	15 (26%)
Coronary artery disease	46 (81%)
Hypertension	44 (77%)
Diabetes	18 (32%)
Chronic lung disease	1 (2%)
Pre-ECMO ejection fraction (%)	24 % ± 14 (5%-60%)
Pre ECMO lactate (mmol/l)	5.85 ± 4.38 (0.6 – 21.2)
Pre ECMO creatinine (mg/dl)	1.6 (1.2)
Pre ECMO RIFEL score for AKI	-
No Risk	13 (22%)
Risk	13 (22%)
Injury	20 (33%)
Failure	15 (26%)

**Table 3:** Indication of cardiac extracorporeal membrane oxygenation.

Indications	N (%)
Acute myocardial infarction	34 (57%)
Post-cardiotomy	13 (22%)
Malignant arrhythmia	4 (7%)
Takotsubo cardiomyopathy	3 (5%)
Drug-induced cardiomyopathy	2 (3%)
Amniotic fluid emboli	1 (2%)
Hypoxic right ventricular failure	1 (2%)

Before placing ECMO, all of the patients with cardiogenic shock were managed medically, whereas pre-ECMO mechanical circulatory support (MCS) was required in 26 patients (43%) (Table 4).

**Table 4:** Cardiac support prior to extracorporeal membrane oxygenation (values are n, %, mean ± standard deviation) CPR: cardiopulmonary resuscitation; ECPR: ECMO-assisted cardiopulmonary resuscitation; ECMO: Extracorporeal membrane oxygenation

Support method	
Percutaneous ventricular assist device (Impella CP)	14 (23%)
Intra-aortic balloon pump	6 (10%)
Cardiopulmonary bypass	5(8%)
Medical management	60 (100%)
ECPR	18 (30%)
Average CPR time (minutes) pre ECPR, Mean ± SD	32 +/- 18

EPCR was performed for 18 (30%) patients, while 42 (70%) received VA ECMO without CPR. Left ventricular venting was utilized for 14 patients (23%) using Impella CP. ECPR was performed in 18 patients (30%) with a mean CPR duration of 32 +/- 18 minutes.

Within this cohort of 60 patients, 51 patients (85%) were placed on ECMO within our facility, while the remaining 9 patients (15%) were cannulated by our mobile ECMO team and subsequently transferred to our hospital. Notably, no device-related complications were seen during these hospital transfers. 

The majority of ECMO cannulations were performed in the intensive care unit (23 patients, 28%), followed by the cardiac catheterization laboratory (21 patients, 35%), the operating room (12 patients, 20%), and the emergency department (4 patients, 7%). Central cannulation was exclusively undertaken in the operating room (14 patients, 23 %); otherwise, peripheral cannulation was more common (46 patients, 77%). For peripheral cannulations in the cardiac catheterization laboratory, fluoroscopy was performed, whereas ultrasound guidance was preferred in other settings to identify femoral vessels for cannulation. Following the procedure, all patients underwent X-rays to ensure the proper placement of the cannula. 

A distal perfusion catheter was placed for all cases except one patient with a known occluded femoral artery. The mean arterial cannula size used in our ECMO patients was 20.2 +/- 1.6 Fr (range 16-23), and for the venous cannula, a size of 25.2 +/- 1.7 Fr (range 23-28) was used. A mean flow rate of 4.26 +/- 0.83 L/min was established and maintained for the initial four hours on VA ECMO.

Out of 60 patients who received ECMO, 10 had less than 24 hours of ECMO run time. Discontinuation for one patient was due to severe disseminated intravascular coagulopathy, while the families of the remaining nine patients opted to withdraw care. The overall duration of ECMO was 5.1 ± 4.3 (0-19) days. Of all the patients, the family withdrew care from 53%, whereas 47% were successfully decannulated after an average of 4.8 ± 3.2 (1-12) days of ECMO. Two patients were transferred to another facility after 24 hours of ECMO support: one at the family's request and the other for further intervention for acute or chronic pulmonary embolism. Both of these patients survived and were later discharged from the hospital.

Our cardiac ECMO program has achieved an overall survival rate of 48% (29/60) alongside an average hospital length of stay for all cardiac ECMO patients of 20 ± 20 days (range 1 to 70 days). Meanwhile, the survival rate to hospital discharge was at 33% (20/60) overall. Upon further examination, the breakdown reveals a hospital survival rate of 36% (15/42) for VA ECMO without ECPR and 28% (5/8) for ECPR (Table 5).

**Table 5:** Survival data (values are expressed n % or mean ± standard deviation) ECMO: extracorporeal membrane oxygenation; ECPR: extracorporeal cardiopulmonary resuscitation; VA ECMO: venoarterial extracorporeal membrane oxygenation

Cardiac ECMO	N (N=60)(%)
ECMO survival	29 (48%)
Hospital survival	20 (33%)
Days on ECMO (all patients)	5.1 +/- 4.31 (0-19)
Days on ECMO (survivors)	4.8 +/- 3.2 (1-12)
VA ECMO except for ECPR	n=42
ECMO survival	22 (52%)
Hospital survival	15 (36%)
ECPR	n=18
ECMO survival	7 (39%)
Hospital survival	5 (28%)

To benchmark our hospital survival outcomes against the ELSO registry data, we categorized our ECMO patients into two groups: one that received VA ECMO without ECPR and another that underwent ECPR. The VA ECMO without ECPR group included 42 patients, and the ECPR group comprised 18 patients, representing 30% of our cohort. The VA ECMO without ECPR group consisted of 31 males (74%) with an average age of 60 ± 12 years, while the ECPR group included 14 males (78%) with an average age of 19 ± 14 years. The hospital survival rates were 36% (15/42 patients) for VA ECMO without ECPR and 28% (5/18 patients) for ECPR (Table 6).

**Table 6:** Comparison of survival data between our institution and reported national average by ELSO. P-value less than 0.05 is considered to be significant ECMO: extracorporeal membrane oxygenation; ECPR: extracorporeal cardiopulmonary resuscitation; ELSO: Extracorporeal Life Support Organization; NA: not available; VA ECMO: venoarterial extracorporeal membrane oxygenation

Comparison of survival data	Our institution	National Average	
VA ECMO except for ECPR	n=42	n=24447	p-value
ECMO survival	22 (52%)	NA	-
Hospital survival	15 (36%)	48%	0.074
ECPR	n=18	n=6650	p-value
ECMO survival	7 (39%)	NA	-
Hospital survival	5 (28%)	30%	0.534

According to ELSO published data, the national average hospital survival of adult cardiac VA ECMO over the past five years was 48%, derived from 24,447 runs. Meanwhile, for ECPR cases, the national average was 30%, based on a total of 6,650 runs [5].

Other complications observed while on ECMO (Table 7). ECMO-related complications were noted as follows: 18% of patients suffered acute limb ischemia (12% at ECMO arterial cannulation side, 5% at Impella cannulation side), One patient experienced acute limb ischemia in both upper extremities, which was more likely due to high vasopressor requirements prior to ECMO rather than due to ECMO or Impella insertion. The most frequent complication was the need for four-compartment fasciotomies in 13% of patients, followed by issues such as cannula malposition, circuit thrombosis, and cannula site bleeding, each occurring in 2% of cases. Non-circuit-related complications occurred in 37% of patients, with bleeding being the most common. The most common site of bleeding was mediastinal bleeding (15%), followed by nasopharyngeal bleeding (5%), and there was one cannulation site bleeding (2%). A total of 86% of patients required blood transfusion during their hospitalization; with the median volume of packed red blood cells (PRBC) transfused was 5 units (IQR 8.5 [2-10.5]), platelets transfused were 0 units (IQR 20, 0-20), fresh frozen plasma (FFP) 0 units (IQR 3.75 [0-3.75]) and cryoprecipitate 0 units (IQR 8.75, 0-8.75). Hemorrhagic and ischemic stroke were seen in 1% and 5% of patients, respectively, during their ECMO duration.

**Table 7:** List of complications (Values are expressed n,%) ECMO: Extracorporeal membrane oxygenation

Complications	-
Acute limb ischemia	11 (18%)
ECMO insertion side	7 (12%)
Impella insertion side	3 (5%)
Non-ECMO/Impella-related	1 (2%)
Fasciotomy	10 (17%)
Cerebral vascular accident	4 (7%)
Anoxic brain injury	2 (3%)
Mediastinal bleeding	9 (15%)
Cannula site bleeding	1 (2 %)
Gastrointestinal bleeding	2 (3%)
Nasopharyngeal bleeding	3 (5%)
Disseminated intravascular coagulopathy	2 (3%)
Cannula malposition	1 (2 %)
Circuit thrombosis	1 (2 %)
Sepsis during ECMO	2 (3%)

## Discussion

VA ECMO stands as the rescue therapy in critically ill patients experiencing cardiogenic shock or as a component part of ECPR and is currently utilized as a part of the resuscitation efforts for both in-hospital and out-of-hospital cardiac arrests. Due to the imperative nature and complexity of the resuscitative intervention with VA ECMO, this intervention demands a competent multidisciplinary ECMO team with a well-defined role to optimize outcomes. Initiating VA ECMO promptly has proven pivotal, reducing the risk of reperfusion injury and enhancing post-cardiac arrest outcomes, including neurological recovery [13-15]. Research indicates that maintaining a shortened CPR time before initiating ECMO proves effective in achieving neurologically favorable survival outcomes [16,17]. Our study's average CPR time was less than 60 minutes, aligning with this beneficial trend.

Contrary to previous notions, recent multicenter trials have shown no significant difference in outcomes between immediate VA ECMO initiation and an early conservative strategy in patients with rapidly worsening severe cardiogenic shock [17]. However, controversy persists, with survival rates on VA ECMO ranging widely (15% to 50%), influenced by the etiology of cardiogenic shock [18-21].

Over the past two years, our community-based ECMO program has developed and adopted a tailored approach and infrastructure to meet the specific needs of our patient population. Before starting our new ECMO program, we engaged in thorough discussions with the administration to secure support and conducted extensive educational sessions on ECMO protocol, cannulation procedure, transportation, and management of ECMO to the shock coordinator, transfer center, emergency transport team, and the nurses. Unfortunately, we cannot provide data before the new ECMO program initiation, preventing a historical comparison.

Implementing a meticulous, time-sensitive, and systematic approach has yielded results comparable to national averages, with a 36% survival rate for cardiac VA ECMO and 28% for ECPR at our institution. While trends favoring national outcomes are evident in terms of survival to hospital discharge, possibly influenced by care withdrawal in 53% of our patients, statistical analysis indicates no significant difference between our institution and the national average (Cardiac VA ECMO p=0.074 and ECPR p=0.534) (Table 6).

Acknowledging the intricacies of ECMO resuscitation, our ECMO program strongly emphasizes identifying key factors for functional survival. This includes prioritizing multidisciplinary communication, selecting suitable patients, integrating a dedicated ECMO team with mobile ECMO emergency services, ensuring capable cardiac ICUs, staffing with dedicated nursing professionals, implementing well-developed protocols, and conducting regular simulations. These efforts collectively contribute to a safe care environment and favorable outcomes, aligning with community needs while optimizing resource utilization and ensuring the sustainability of our program.

This present study's key limitations include the small number of patient population, regional variations, resource constraints, comorbidities, and varying illness severities at presentation. Despite the above limitation, our study demonstrates that once optimized protocols, cannulation standards, and management by a designated management team are in place, the outcomes of our community hospital's VA ECMO program align with those of the national average.

## Conclusions

Once protocol, cannulation standards, and management were optimized in the community hospital, the results of VA ECMO at a community hospital system with cardiac surgery capability were not inferior to national results.
